# Recycling existing drugs for cancer therapy: delivering low cost cancer care

**DOI:** 10.3332/ecancer.2014.ed40

**Published:** 2014-07-10

**Authors:** Pan Pantziarka, Linda Cairns

**Affiliations:** 1 Anticancer Fund, Brussels, Belgium; 2 The George Pantziarka TP53 Trust, London, UK; 3 Science Editor, ecancermedicalscience, European Institute of Oncology, Milan, Italy

Cancer is one of the leading causes of death today and is only set to worsen as its incidence continues to rise worldwide. The development of novel and effective anti-cancer drugs is a lengthy, extremely costly and inefficient process and many potential compounds are eliminated at the preclinical stages and thereafter many still never make it to the market. The cancer medical community, probably more than any other, understands the urgent need for more effective therapies as the current high cost of cancer care is unsustainable. Drug repurposing or drug repositioning is the application of established drugs to new indications and represents an increasingly promising way to speed up the development of treatments for diseases that do not respond well to standard therapies.

The cost of cancer drugs has more than doubled in the past decade. Of the 12 cancer drugs approved in 2012 by the FDA for cancer 11 were priced at more than $100,000 per patient per year. The cost of Gleevec (Imatinib), the enormously profitable Novartis drug for the treatment of chronic myelogenous leukemia, has risen from £18,000 per patient per year to around £21,000 in the UK, and from $30,000 to $92,000 in the US. This is despite the fact that all the research costs were covered by the original price, and the number of patients treated and the length of time they are on the drug have both vastly increased because of the drug’s success. Cheap generic versions are available and will hopefully enter the market as early as 2015 when the main patent on Gleevec expires. In fact the Indian Supreme court overturned a recent attempt by Novartis to extend the patent and block the use of generic versions of Imatinib.

Drug repurposing is a strategy with fascinating potential for cutting the cost of cancer care as well as significantly affecting patient outcomes, with two of the most notable examples being Thalidomide and Sildenafil. Thalidomide was commonly used in the late 1950s as a sedative in pregnant women, but was later found to be associated with serious birth defects. Today, it is used to treat multiple myeloma due to its anti-angiogenic activity. Sildenafil (Viagra) was being developed by Pfizer to treat high blood pressure when its ability to ‘treat’ erectile dysfunction was identified as a side-effect, resulting in a complete shift in marketing strategy.

Repurposing of approved or abandoned drugs for cancer represents an opportunity to rapidly advance to patients promising drug therapies by capitalizing on existing data and experience. It can take advantage of previous research and development efforts, and detailed information about the drug formulation and safety is usually available, meaning that it can be ready for clinical trials much faster than a brand-new drug.

While our understanding of the biology and genetics of cancer has increased dramatically in recent decades, the pace of discovery, development and registration of new drug therapies for cancer has not. Searching for new treatments for cancer among drugs that were already available for other diseases relies on a much greater knowledge of the basic nature behind how these established agents work. This will require new thinking, new approaches, and new collaborations.

In fact Cancer Research UK has reached an agreement with AstraZeneca to take an experimental drug, originally designed for asthma, into a clinical trial to treat kidney cancer. In June 2013, the NIH announced funding for nine cooperative agreements between academic research groups with selected industry. The goal is to identify molecules that have already undergone significant research and development by the pharmaceutical industry to more quickly advance new treatments for patients.

A recent paper in Cancer Research [1] demonstrated the power of bioinformatics-based drug approaches to rapidly repurpose approved drugs and identified a novel class of molecules, Tricyclic Antidepressants, as Inhibitors of Small Cell Lung Cancer, a cancer for which no effective novel systemic treatments have been identified in several decades.

The Repurposing Drugs in Oncology (ReDO) project, an international collaboration between researchers working for not-for-profit patient-centred organisations in Europe and the United States, aims to accelerate the repurposing of non-cancer drugs for new indications in oncology. To do this it aims to identify the most promising candidate drugs; to summarise the often dispersed pre-clinical and clinical data; to explore potential combination therapies with existing anti-cancer or other repurposed drugs; and to work with investigators to develop clinical trials using these combinations.

Many of the candidate drugs identified by the ReDO project are low-cost and/or generic drugs that have well-characterized pharmacokinetics and toxicity profiles at clinically relevant doses and have direct clinical evidence of efficacy – often from small trials or from published case reports. Example candidates include mebendazole, nitroglycerin, cimetidine, clarithromycin, diclofenac and itraconazole. ReDO hopes to make known their potential clinical benefit in the treatment of cancer and to bring these drugs to patients as quickly as is possible. Ideally, multiple foundations should support investigator-driven pivotal trials to confirm the efficacy of these products or product combinations.

## Conclusion

The exorbitant cost of cancer drugs can be the difference between living and dying for many patients – a solution must be found to bring down the cost of cancer care. Drug repurposing and generic versions are an obvious way forward, and significant resources should be directed into these areas of research. To this end, ecancer will be publishing a series of articles on drug repurposing, submissions are welcome on the repositioning of any kind of drug for cancer treatment.
